# Psychological Symptoms in COVID-19 Patients: Insights into Pathophysiology and Risk Factors of Long COVID-19

**DOI:** 10.3390/biology11010061

**Published:** 2022-01-02

**Authors:** Angel Yun-Kuan Thye, Jodi Woan-Fei Law, Loh Teng-Hern Tan, Priyia Pusparajah, Hooi-Leng Ser, Sivakumar Thurairajasingam, Vengadesh Letchumanan, Learn-Han Lee

**Affiliations:** 1 Novel Bacteria and Drug Discovery (NBDD) Research Group, Microbiome and Bioresource Research Strength (MBRS), Jeffrey Cheah School of Medicine and Health Sciences, Monash University Malaysia, Subang Jaya 47500, Malaysia; angelthye.yunkuan@monash.edu (A.Y.-K.T.); jodi.law1@monash.edu (J.W.-F.L.); loh.teng.hern@monash.edu (L.T.-H.T.); priyia.pusparajah@monash.edu (P.P.); ser.hooileng@monash.edu (H.-L.S.); 2 Clinical School Johor Bahru, Jeffrey Cheah School of Medicine and Health Sciences, Monash University Malaysia, Johor Bahru 80100, Malaysia

**Keywords:** COVID-19, mental health, anxiety, depression, post-traumatic stress disorder (PTSD), SARS-CoV-2, ACE-2, immune inflammatory signaling

## Abstract

**Simple Summary:**

The coronavirus can elude the immune response, potentially spreading to cells other than the respiratory tract’s epithelial cells. The neuro-invasive potential of certain coronaviruses has been observed in the severe acute respiratory syndrome (SARS) and Middle East respiratory syndrome (MERS). Based on past outbreaks, including SARS, MERS, and current reports of neuropsychiatric complications following COVID-19, many survivors may be at risk of a number of neuropsychiatric sequelae. Mounting evidence has shown the presence of mental health implications in COVID-19 survivors. This review shows that psychological symptoms including anxiety, depression, and post-traumatic stress disorder (PTSD) have an association with post-COVID-19 infection. The exact cause of these psychiatric sequelae is yet to be determined, but it seems to involve environmental, psychological, and biological factors. Although there are variations in terms of risk factors and the prevalence rate of these psychological symptoms, risk factors including the female gender and having a history of psychiatric disorders appears to be consistent a across few studies, and there are studies showing a higher prevalence rate among post-COVID-19 survivors than among the general population. A therapeutic intervention commonly used to alleviate these psychological symptoms are psychotropic medications, but probiotics could be a safe adjunctive treatment to improve these symptoms.

**Abstract:**

There is growing evidence of studies associating COVID-19 survivors with increased mental health consequences. Mental health implications related to a COVID-19 infection include both acute and long-term consequences. Here we discuss COVID-19-associated psychiatric sequelae, particularly anxiety, depression, and post-traumatic stress disorder (PTSD), drawing parallels to past coronavirus outbreaks. A literature search was completed across three databases, using keywords to search for relevant articles. The cause may directly correlate to the infection through both direct and indirect mechanisms, but the underlying etiology appears more complex and multifactorial, involving environmental, psychological, and biological factors. Although most risk factors and prevalence rates vary across various studies, being of the female gender and having a history of psychiatric disorders seem consistent. Several studies will be presented, demonstrating COVID-19 survivors presenting higher rates of mental health consequences than the general population. The possible mechanisms by which the severe acute respiratory syndrome coronavirus 2 (SARS-CoV-2) enters the brain, affecting the central nervous system (CNS) and causing these psychiatric sequelae, will be discussed, particularly concerning the SARS-CoV-2 entry via the angiotensin-converting enzyme 2 (ACE-2) receptors and the implications of the immune inflammatory signaling on neuropsychiatric disorders. Some possible therapeutic options will also be considered.

## 1. Introduction

Coronaviruses—single stranded RNA viruses—have caused several outbreaks in recent years, with the most notable one being the recent COVID-19 pandemic, caused by severe acute respiratory syndrome coronavirus 2 (SARS-CoV-2). The severe acute respiratory syndrome (SARS) and the Middle East respiratory syndrome (MERS) were also caused by coronaviruses [1]. SARS-CoV-2 has spread rapidly and widely across the globe in a matter of months [2]. According to data from John Hopkins Coronavirus Resource Center, as of 19 October 2021, the COVID-19 global pandemic has caused 4,912,316 deaths and 241,513,188 confirmed cases worldwide [3]. Initially, COVID-19 consisted of four variants of concern (VOC), which includes the Alpha (B.1.1.7), Beta (B.1.351), Delta (B.1.617.2), and Gamma (P.1) variants [4]; however, recently, on 26 November 2021, the World Health Organization (WHO), designated the variant B.1.1.529 a VOC, named Omicron [5]. This new VOC was first reported to the WHO in South Africa on 24 November 2021 [6]. Omicron possesses several mutations that impact its behavior. Despite the increase in COVID-19 cases associated with the Omicron variant in areas of South Africa, it is not yet certain if this variant is indeed more transmissible. In terms of the severity of the disease, although preliminary data found an increase in the hospitalization rate in South Africa, more time is needed to better understand if Omicron results in a more severe disease than the other variants [5].

Although the pathogenesis, clinical characteristics, epidemiology, and complications of acute-phase COVID-19 patients have been explained [7,8], the long-term consequences remain unclear [9]. However, it may be possible to extrapolate these based on the experience from SARS and MERS, given the phylogenetic similarities between the pathogenic coronaviruses causing these infections [10]. SARS-CoV-2 has a 79% overlap of genomic sequence identity with the severe acute respiratory syndrome coronavirus 1 (SARS-CoV-1), and 50% with the Middle East respiratory syndrome coronavirus (MERS-CoV) [11,12]. Moreover, both SARS-CoV-1 and SARS-CoV-2 use the angiotensin-converting enzyme 2 (ACE-2) as the host cell receptor, but with SARS-CoV-2 having a greater affinity for ACE-2 [10]. Given their similarities, it is possible that the current COVID-19 pandemic could have a similar trend as to the past SARS and MERS outbreaks.

It is well known that the SARS-CoV-2 infection has a classical respiratory virus-like clinical manifestation, with more than 80% of patients experiencing a mild-to-severe and self-limiting infection [1]. The host immune system will recognize the virus and activate the innate immune response, the T-cell and B-cell immunity, and antiviral neutralizing antibody response in COVID-19 patients [13]. This is because when there is an intracellular infection, the innate immune system will actively induce cell self-destruction, such as programmed cell deaths [14,15,16,17], apoptosis [14,15], necroptosis [16], and pyroptosis [17], to stop the intracellular infection. The innate immune system can reuse the nutrition from the degradation of these destroyed cells and the microorganisms in these cells to rebuild the tissue cells.

Typically, the innate immune system releases a rapid antiviral response through I/III IFN, cytokines (IL-1, IL-18, and IL-6), and chemokines (CCL2 and CCL7) to inhibit virus replication [18]. The CD8+ cytotoxic T-cell kills the infected cell and slows down the infection. Then, the CD4+ helper T-cells will stimulate B-cells to produce antibodies (IgM and IgG) specific to the virus. Once the infection is over, the number of T-cells and B-cells will decline but remain as memory cells if the host reencounters the same virus. In most cases, the viral and bacterial infections are thus self-limiting [19,20]. However, severe cases of common illnesses are caused by an overreaction from the disrupted host immune response [21,22,23]. As quoted by Sir William Osler more than 100 years ago, “With the exception of few cases, the patient happens to die from their own body’s response to the infection rather than from it” [21]. Hence, instead of worrying about the SARS-CoV-2 viral infection, individuals should pay more attention to their pre-existing health conditions such as obesity, diabetes, and metabolic syndromes, which have a high connection with the interruption of the immune system.

Post-infectious sequelae of viral infection often include damage to many different organs via a variety of pathological mechanisms—with the brain being one of the often-targeted organs [24]. SARS-CoV-2 has been shown to affect the respiratory system and the nervous, renal, and cardiovascular systems [25]. Along with the increase in cases of COVID-19, recognition of the mental health consequences of infection has also increased [26,27,28]. This would be consistent with previous outbreaks, including SARS and MERS, which are linked with long-term neuropsychiatric implications [29,30], as seen in a meta-analysis showing that approximately one-third of SARS and MERS survivors have psychological conditions such as anxiety, depression, and post-traumatic stress disorder (PTSD) persisting 6 months after discharge from hospital [31]. During the earlier SARS outbreak, previous data demonstrated that the coronavirus could result in prolonged mental disorders with long-lasting neuropsychiatric repercussions [32,33]. Psychiatric symptoms reported by SARS survivors include depression, PTSD, obsessive–compulsive disorder (OCD), and panic disorder at 1 to 50 months follow-up [24,32,34]. According to recent emerging data, COVID-19 infection is linked with confusion, delirium, depression, fatigue, insomnia, PTSD, anxiety, and obsessive–compulsive symptoms, which were picked up during short-term follow-ups after clinical recovery or acute viral infection, with the severity of systemic inflammation during the acute infection being proportional to the severity of psychiatric symptoms after virus clearance, which affects COVID-19 survivors’ quality of life [35,36,37,38].

We aim to discuss the possible causes, prevalence, and risk factors of COVID-19-associated psychological effects, particularly anxiety, depression, and PTSD, which occur during the infection and post-infection. Currently, data on COVID-19-associated psychological effects is limited, so we also utilize data from SARS and MERS infections to speculatively predict the psychological implications of COVID-19. We also discuss the possible mechanisms of how SARS-CoV-2 enters the brain and affects the central nervous system (CNS), leading to long-term psychological consequences. Then, we review evidence of psychological sequelae of COVID-19. This is vital, given the potential public health implications, as the number of individuals psychologically affected will almost certainly increase as COVID-19 infections increase. Even if the fraction of infected individuals affected psychologically is low, considering the impact of the pandemic, this could still have significant implications. A proper understanding of COVID-19-related psychological consequences will enable suitable and efficient mental health care plans and psychological rehabilitation to be provided in a timely manner to these COVID-19 survivors to improve individual functioning. Lastly, we will discuss probiotics as an adjunctive treatment.

## 2. Research Methodology

**Figure 1:**
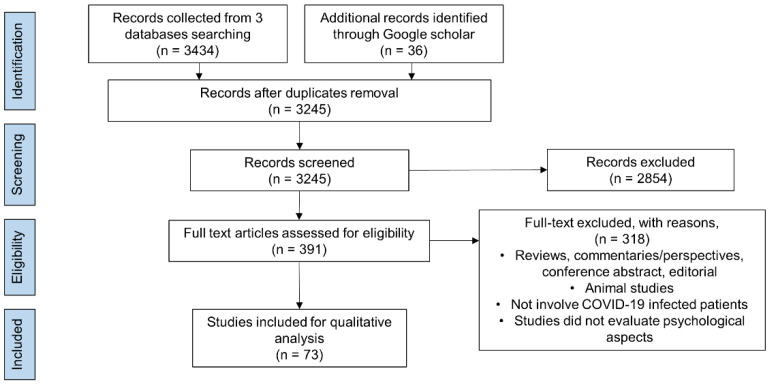
PRISMA flow diagram of the study search and selection process.

The current narrative review searched for COVID-19 impacts on the psychological aspects in patients with COVID-19 using the “preferred reporting items for systematic review and meta-analysis” (PRISMA) statement (Figure 1). The literature search was performed across three databases, PubMed, Web of Science, and Ovid Medline, using the following keywords to search for relevant articles: “COVID-19” OR “long COVID” AND “depression” OR “psychological” OR “anxiety” OR “cognitive” OR “neuropsychiatric” OR “stress” OR “mental health”. Google Scholar was used to search for additional literature not captured in the aforementioned databases. We included all articles related to the psychological sequelae among the confirmed COVID-19-infected patients. Articles written in English were all included in this search. To ensure the reliability of this narrative review, two authors independently screened the titles and abstracts yielded by the search against inclusion criteria. The full-text content of the included articles was reviewed by authors to determine their relevance, while any disagreement was resolved through discussions. As a result of the reviews, 73 studies that met the inclusion criteria were included in this narrative review.

## 3. Cause, Prevalence, and Risk Factors of COVID-19-Associated Psychological Effects

Little is known about the cause, prevalence, and risk factors of COVID-19’s prolonged psychological effects. Although the body of literature around psychological symptoms in the aftermath of COVID-19 infection is growing, the results available are mostly from studies based on surveys or are self-reported by patients, and therefore need to be interpreted with caution. Overall, however, the data suggest that the problem is significant and provide insights into some possible reasons for why COVID-19 may have psychological effects. Psychological manifestations could be related to those virus-infected individuals who are worried about the stigma [39], the outcome of the illness [26], traumatic memories of severe illness or amnesia [40], the psychological reactions after contracting COVID-19, and the associated medical interventions [1]. However, it could also affect both infected and uninfected individuals due to experiences related to the pandemic, including social isolation [41], anxiety [42], financial difficulties and unemployment [43], and stress in both essential workers and health care workers [44]. COVID-19-related psychiatric disorders are likely multifactorial due to a combination of environmental, psychosocial, and biological factors resulting from this global pandemic [45].

Patients who recovered from SARS and MERS infections have shown a high prevalence of anxiety, depression, and PTSD [29]. In a meta-analysis, Rogers et al. found that 27–41% of cases reported neuropsychiatric symptoms during acute SARS and MERS infections, including anxiety, depressed moods, confusion, insomnia, and even a minority with psychoses and steroid-induced mania [1,29]. Long-term neuropsychiatric effects were also found in >15% of SARS and MERS survivors, including emotional lability, recall of traumatic memories, fatigue, sleep disorders, and memory impairments [29]. In another study, where 42.5% SARS survivors experienced ≥1 active psychiatric illness, 54.5% were found to experience PTSD, while 39% had depression, 36.4% had a pain disorder, 32.5% had a panic disorder, and 15.6% had OCD, which is a huge increase compared to the 3.3% pre-infection prevalence of any psychiatric diagnoses [32]. Given the similarities between SARS and SARS-CoV-2 and MERS, it seems fair to speculate that COVID-19 infections might follow a similar trend.

Concerning COVID-19, the prevalence of mental health problems in the general public and health care workers ranged from 20–36% [46]. In a study conducted in Wuhan, COVID-19 patients indeed demonstrated a higher prevalence of anxiety (38.5%) and depression (35.9%) [47]. The prevalence of post-infection anxiety ranges from 6.5–63% [48]. One study showed that at 2–4 months post-hospitalization, COVID-19 survivors showed an anxiety rate between 14–47.8% [49,50,51]. On the other hand, in studies involving both hospitalized and non-hospitalized patients, the prevalence rate for depression ranges between 12–48% [52,53]. In terms of PTSD, at 1–3 months post-COVID-19, its prevalence rate ranges from 12.1–46.9% [48]. Furthermore, there are findings of a 10% prevalence rate 6 weeks post-discharge [54], a 36.4% rate 2 months post-hospitalization [55], and at 3–4 months post-hospitalization, 5.9% of survivors had symptoms consistent with severe PTSD; 11.3% had moderate symptoms and 25.6% had mild symptoms [56]. There is a wide range in the prevalence of various mental health symptoms among studies. This could be due to variations in assessment methods and the instruments used to measure these outcomes, different samples or differences among countries in the implications of spiritual or cultural beliefs to manage the psychological consequences of coronavirus disease, and the different time frames for follow-ups [45,57,58]. Thus, these findings should not be generalized, and more studies, such as observational studies and prospective cohort studies, are needed.

There appears to be slight variations in the risk factor profile for the different psychiatric manifestations associated with COVID-19. Risk factors associated with anxiety include being female [59], illness severity [9,60], medical comorbidities [61], having close relatives with COVID-19 [62], perceived discrimination, the greater total number of symptoms post-discharge, living with children, a death of a family member from COVID-19 [47,60], decreased quality of life and persistent dyspnea [50], reduced sense of smell [63], history of psychiatric illness, stigma of COVID-19 infection [64,65], poor social support, use of tobacco in the past three months [66], and being of a younger age [67], while the risk factors for depression are being of the female gender [9,35,47,68], having a history of psychiatric illness, stigma of COVID-19 infection [65], poor social support [66], perceived discrimination, living with children, having infected family members, smoking, higher education level, greater total number of symptoms [47,63], baseline immune response [69], illness severity [9,63], reduction in the sense of smell, and being of an older age [63]. Studies found that at 2–3 months post-hospital discharge, the rate of moderate-to-severe depression experienced was between 10–42% [35,49,50,51,55,62,63,64,70]. This group of patients with more severe depression had higher COVID-19-related perceived stigma [63,71], were quarantined after hospitalization [62], and had a history of psychiatric manifestation [35,64]. Lastly, risk factors for PTSD includes history of psychiatric illness, stigma of COVID-19 infection, total duration of isolation [65], fatigue, chest distress and cough that arise due to COVID-19, perceived discrimination, illness severity, living with own children, and death of a family member [63]. Findings from one Israel study found that feeling socially disconnected predicted the presence of PTSD one month after hospitalization [72]. Interestingly, a China study found trauma exposure, negative media reports, and lower perceived social supports were risk factors for all three—anxiety, depression, and PTSD [73]. Nonetheless, there seems to be some variation in the findings of risk factors across different study groups from different countries (China, Italy, Bangladesh, Switzerland, United States, Spain, and Korea).

A few risk factors seemed consistent over a range of neuropsychiatric complications. Studies found females having a history of psychiatric diagnosis [2,59,74,75,76], and those with psychopathological symptoms a month post discharge, suffered more in all psychopathological domains [2]. Overall, female gender tends to be a consistent risk factor for various psychological disorders, with several studies showing females post-COVID-19 have a 2.2–2.5-times higher chance of developing a psychiatric morbidity [37,48,77,78,79]. This finding is consistent with SARS studies, where female SARS survivors were also at a higher risk of depression, anxiety, and stress levels [80]. In fact, another study even found that women were more represented among dead COVID-19 patients with the common mental disorder than men [81]. However, one Chinese study found no significant difference in anxiety or depressive symptoms among males and females [82]. Although having a history of psychiatric disorder seems to be associated with increased severity of post-COVID-19 psychiatric symptoms [35,37], even those without any history of mental health morbidity (74%) did report symptoms of depression and anxiety post-COVID-19 [77]. This finding is consistent with two meta-analyses of survivors of previous SARS and MERS outbreaks [29,31], with a third of patients reporting ≥1 psychological impairment (anxiety, depression, PTSD) >6 months post-discharge [31]. On the other hand, age as a risk factor had inconsistent findings. A multivariable analysis from China showed females and those with a severity scale of 5–6 were associated with a higher risk of depression or anxiety, but age had no significant association with both depression and anxiety [9]. Although three other studies demonstrated consistent data that no association was found between age and psychological symptoms [24,48,51,78], at least four other studies showed an inverse relationship [35,37,48,51,77,83].

## 4. Psychiatric Sequelae of COVID-19

Neuropsychiatric consequences may occur due to disease or brain damage due to direct CNS infection or indirect effects on the CNS via an immune response or medical therapy [29]. COVID-19 could negatively impact the cognition and quality of life of COVID-19 survivors [84]. Months after the initial COVID-19 infection, individuals continue to experience a range of psychiatric symptoms [85]. Surveys have reported that the acute psychiatric manifestations of COVID-19 are increased depression, anxiety, and stress [42]. The long-term psychiatric manifestation of COVID-19 is not known yet, but their prolonged effects could be speculated based on evidence from SARS and MERS, as well as understanding COVID-19’s effects on the central nervous system (CNS) [1]. Some SARS survivors showed persistent mental issues at a 1-year follow-up, even though their physical conditions had improved [80,86]. Some continued experiencing mental consequences that persisted and stayed clinically significant at up to 4 years of follow-up [32]. Few prospective studies have shown that symptoms of long COVID-19 can persist 3 months [87], 5 months [88], 6 months [89,90], and even up to 12 months [91,92] post-hospitalization. Several studies have also shown the presence of psychiatric manifestations post-COVID-19 infection. Here we will focus on anxiety, depression, and PTSD post-COVID-19. According to an analysis, the prevalent symptoms of PTSD in post-COVID-19 patients include a difficulty concentrating, sleep issues, feeling distant from people, and intrusive thoughts [63]. In fact, sleep disturbance is common in critically ill patients up to 1 year following hospital discharge, with the prevalence ranging between 10% and 60% at 6-month intervals [93,94]. However, given the lack of available data, this association with the current COVID-19 pandemic can only be hypothesized [94].

In addition to general mental health issues [95,96], the rates of anxiety, depression, post-traumatic stress symptoms, sleep difficulties, and fatigue rates in COVID-19 survivors have increased [45], with anxiety and PTSD more often affecting those who were admitted to ICU than wards [37,51,77,97]. In a UK study, compared to contemporaneous patients diagnosed with various respiratory tract infections, patients who had recovered from COVID-19 infections showed higher anxiety and mood disorder rates at 6 months post-diagnosis [98]. A Chinese study also showed that a significant percentage of patients discharged after 6 months had depression/anxiety (23%) and sleep abnormalities (26%) [9]. Worryingly, reports of anxiety or depression were higher at 12 months than at 6 months [99]. In addition, a study in the US involving 999 participants, with an average age of 45 years, 77% of whom being white and 49% male, found prolonged COVID-19 to be present in 25% of participants, the symptoms of which lasted for months post-diagnosis and were disabling, interfering with household or work responsibilities [100]. The most common symptoms found were anxiety, fatigue, headache, and brain fog, consistent with other studies [9,100,101,102,103]. Moreover, Huang et al. showed that depression, anxiety, sleep difficulties, fatigue, and muscle weakness were common 6 months after a COVID-19 infection [9]. In agreement with Huang et al., a study by El Sayed et al. also found a high score of fatigue in post-COVID-19 patients [104]. Additionally, this is consistent with a SARS long-term follow-up study where 33% of survivors experienced significant declines in their mental health 1-year post-infection [86]. The high rates of PTSD seen in COVID-19 patients may have a similar trend to the SARS outbreak, as a study showed that 25% of SARS survivors experienced significant PTSD symptoms after 30 months [105]. However, one study did show no differences in depression, anxiety, sleep, and fatigue scores between negative- and positive COVID-19 respondents [100].

Some other published studies include a prospective cohort study in Milan with a sample size of 402, which found that at 1-month post-COVID-19, 55.7% participants scored ≥1 psychopathological dimension (depression, anxiety, PTSD, and OCD), 36.8% in two, 20.6% in three, and 10% in four [35]. A single-center study in Spain on COVID-19 survivors showed that, out of 179 patients, several had anxiety, depression, and PTSD at 29.6%, 26.8%, and 25.1%, respectively, two months post-COVID-19 [78]. Another cohort study in Milan showed COVID-19 survivors were still clinically depressed 3 months post-discharge, while other symptoms which are more associated with acute psychological stressors, for instance, anxiety, insomnia, and PTSD, reduced over time [2]. This is somewhat consistent with a recent systematic review and meta-analysis of longitudinal cohort studies comparing mental health before versus during the COVID-19 pandemic in 2020. The studies found that, compared to pre-pandemic, there was an overall increase in mental health symptoms from March–April 2020, which dropped significantly over time and became non-significant from May–July 2020 [106]. Nevertheless, one out of eight patients still report significant PTSD symptoms at one-month post-discharge [63].

Coronavirus is an opportunistic virus which can elude the immune response, potentially spreading to cells other than the respiratory tract’s epithelial cells. The neuro-invasive potential of certain coronaviruses can be observed in SARS and MERS [107,108,109]. Based on past outbreaks, including SARS, MERS, and current reports of neuropsychiatric complications following COVID-19, many survivors may be at risk of a number of neuropsychiatric sequelae [1]. In fact, roughly 30–40% of patients reported clinically significant anxiety and depression following a COVID-19 infection, similar to patients with previous severe coronavirus infections [10,29,32,35,80,110]. A study showed that 41.3% of patients in Iran, and a third of COVID-19 patients in Italy, experienced anxiety and depression post-discharge [51,111]. In contrast, another study from China showed that, at 6 months post-discharge, 23% of patients experienced anxiety or depression [9]. Findings from the Chinese study were somewhat similar to findings from a Korean study, where long-term psychological sequelae were identified and made up ≥20% of all sequelae [112]. Moreover, based on data from 54 healthcare organizations in the United States involving a total of 62,354 COVID-19 survivors, the incidence of first and recurrent psychiatric illness between 14–90 days post-diagnosis is 18.1% [113]. Within 90 days after a diagnosis of COVID-19, the estimated overall probability of a diagnosis of a new psychiatric illness is 5.8% among a subset of 44,759 patients with no history of psychiatric illness. Compared to matched control cohorts of patients diagnosed with influenza and other respiratory tract infections, these values were all much higher [10,113]. In addition, some studies showed hospitalized clinically stable COVID-19 patients reported higher rates of PTSD (96.2%) [114], depression (60.2%), and anxiety (55.3%) than normal controls [115]. In contrast, another study in China showed that the prevalence rates of clinically significant depression, anxiety, and PTSD symptoms for hospital-discharged COVID-19 patients are 19%, 10.4%, and 12.4%, respectively [63,116]. Although there is a huge difference between studies, these anxiety and depression rates are much higher than the rates found in the normal general adult population in China [117]. Additionally, an Ethiopian study carried out amid the COVID-19 pandemic also found that, among chronic medical patients, the prevalence rate of anxiety (61.8%) and depression (55.7%) was higher than the prevalence rate of anxiety (32%) and depression (5.73%) before the COVID-19 pandemic [66].

## 5. Pathophysiology of COVID-19’s Psychological Effects

Although the pathophysiological mechanism of SARS-CoV-2 on different physiological systems has not been fully understood [1] and the prolonged or long-term consequences of neuropsychiatric manifestations post COVID-19 is yet unknown, we can speculate its pathophysiological mechanisms and implications from what is known about other coronavirus subtypes. Coronaviruses mainly affect the upper respiratory tract, but they have been found in the cerebrospinal fluid and the brains of infected individuals [118]. SARS-CoV-2 has been detected via gene sequencing in the cerebrospinal fluid of viral encephalitis-diagnosed patients, confirming its neuro-invasive potential [119]. An autopsy series also shows that SARS-CoV-2 possibly leads to brain parenchyma and vessels alteration via effects on blood–brain and blood–cerebrospinal fluid barriers that drive inflammation in supportive cells, brain vasculature, and neurons [10,120,121]. A study also provides a detailed characterization of the functional neuroimaging correlates of long COVID-19 symptoms and subtypes, assisting in the development and implementation of effective treatments for these conditions [122].

Coronavirus can induce psychopathological sequelae indirectly through an immune response or by a direct viral infection of the central nervous system (CNS) [24]. Mechanisms that contribute to COVID-19’s neuropathology involve a variable combination of direct viral infection, neuroinflammation, severe systemic inflammation, neurodegeneration, and microvascular thrombosis [10,123,124,125,126]. Several other studies also hypothesized that viral infections could prompt chronic inflammation and aberrant immune responses, resulting in long-lasting neuropsychiatric symptoms involving affective, behavioral, and cognitive symptoms over fluctuating periods post-infection [29,30,32,127].

### 5.1. SARS-CoV-2 Entry into the Brain/CNS

Coronaviruses can damage the nervous system by the direct invasion of the CNS, in which the virus can enter via the blood-circulation pathway, neuronal pathway, and by binding to angiotensin-converting enzyme 2 (ACE-2) receptors [1,24]. The mechanisms of injury include direct infection injury, hypoxic injury, and immune injury [1,24]. ACE2 receptors are expressed in various organs, including the lungs, kidney, heart, venous endothelial cells, testicles, small intestinal enterocytes, and even the brain [128,129]. The coronavirus can spread around the body through the bloodstream and across vascular beds of various organs via the disruption of ACE2-bearing endothelial cells [130]. The hypotheses used to explain the entry of SARS-CoV-2 into the CNS include neuronal retrograde transport, hematogenous dissemination, and passage through the nasal cavity across the cribriform plate that supports the olfactory bulb [119]. Wu et al. suggested that the SARS-CoV-2 virus enters the brain directly as its spike protein binds to ACE-2 receptors in capillaries, disrupting the blood–brain barrier (BBB) [24]. As they cross the BBB, there will be increased binding to high density ACE-2 receptors present on neurons. In recovered COVID-19 patients, SARS-CoV-2 can lie latent in the neurons, resulting in demyelination and neurodegeneration, causing greater risk of long-term effects [131]. Although the literature does reveal some differences between the behaviour of SARS-CoV-2, as compared to SARS and MERS, in the brain, they have sufficient similarities, so it seems safe to suggest that the neurotropic capacities of coronaviruses, which allows the evasion of the host’s immune system response and the ability to remain latent in the neurons, is likely to be the root cause of SARS-CoV-2-related long- and short-term neuropsychiatric sequelae [1,24].

### 5.2. Implications of Immune Inflammatory Signaling on Neuropsychiatric Disorder

Cognitive-behavioral changes have shown a correlation with elevated inflammatory markers, which correlates with an increased level of immune activation [132,133,134]. This has significant implications in COVID-19 infections where systemic inflammatory responses are a key part of the pathophysiology of infection. This implies that even without direct viral infiltration into the CNS, the involvement of peripheral cytokines in the host’s antiviral response could cause neuroinflammatory responses and/or compromised the blood–brain interface’s integrity, resulting in peripheral immune cell transmigration into the CNS and the disruption of neurotransmission, possibly inducing psychiatric symptoms [135,136]. Cytokine dysregulation (particularly transforming growth factor-β (TGF-β), Tumor Necrosis Factor (TNF)-α, Interleukin (IL)-1β, Interferon (IFN)-γ, IL-6, and IL-10) are known to be associated with psychiatric disorders [35,137,138,139,140,141,142], and have been shown to be elevated in patients with COVID-19. This, then, suggests the possibility of a link between COVID-19 infections and neuroinflammation, peripheral immune cell invasion into the CNS, hypothalamic-pituitary (HPA) axis dysfunction, BBB disruption, neurotransmission alteration, microglia activation, oxidative stress, and indoleamine 2,3-dioxygenase 1 (IDO) activation, all of which represent the pathways linking the psychopathological mechanism and the immune system, possibly underpinning the etiology of psychiatric disorders [135,141,143,144,145].

The local and systemic production of chemokines, cytokines, and other inflammatory mediators are induced as an immune response to coronaviruses [146]. Coronavirus binds directly to ACE-2 receptors in the respiratory epithelial cells, potentially resulting in a cytokine storm that causes widespread inflammation, resulting in multi-organ damage and immune-mediated encephalopathy that exhibits convulsions and delirium [1,147]. This cytokine storm causes an increase in T-helper (Th)-1 cytokines, including TNF-α, IL-1β, CCL2, CXCL10, IL-6, and IFN-γ, and Th-2 cytokines, including IL-10, IL-4, and IL-1 receptor antagonists in the serum of COVID-19 patients [2,148]. Interestingly, the cytokine-release syndrome, or the inflammatory response related to COVID-19 infections [149,150,151,152], possibly synergizes with a stressor-related inflammatory response to prolong and increase the post-viral symptoms [100].

In terms of depression, the association between depression and inflammation is well described and may account for some elements of the psychiatric morbidity [153]. A possible mechanism for depressive psychopathology could be the increased inflammatory/immune setpoints with increased circulating biomarkers of inflammation that are seen in mood disorders without any triggering factors [2,154,155,156]. Some studies have shown that COVID-19 severity is associated with increased levels of IL-6 [149,150,151,157,158], which have been linked to changes in activity in the subgenual cingulate cortex and depression [159,160]. Additionally, consistent across several studies were the elevated levels of cytokines CCL2, IL-1β, IL-10, IL6, TNF-α, and the transforming growth factor-β (TGF-β) in depressed patients, relative to healthy controls [2,137,154,161,162,163]. Last but not least, we consider the systemic immune-inflammatory index (SII), an objective marker based on peripheral lymphocyte, platelet, and neutrophil counts, thus providing a means of expressing the balance between immune response status and host systemic inflammation, as these cells have roles in many pathways involved in the inflammatory/immune response [164].

## 6. Possible Therapeutic Options

In the presence of neuropsychiatric symptoms following long COVID-19, probiotics should be considered as an adjunctive treatment. Probiotics are one of the potential adjunctive treatments for psychiatric sequelae among COVID-19 survivors, other than the conventional use of psychotropic medications. Within the last decade, numerous research and clinical trials have been conducted to determine the effects of probiotics on mental health, and clinical trials have proven efficacy for improving mental illness [165]. A systematic review and meta-analysis have shown that probiotic administration was associated with lower depression and anxiety, relative to the placebo group at the end of treatment [166]. On the other hand, a clinical study conducted by Allen et al. involving 22 healthy volunteers revealed that 4 weeks of administration of *Bifidobacterium longum* 1714 (1 × 10^9^ colony-forming units per stick) strains improved cortisol production and hippocampal-dependent visuospatial memory. It also reduced subjective anxiety and daily stress [167,168].

There is currently research studying the application of probiotics to prevent and treat COVID-19 [169]. Additionally, a study has also hypothesized that the homeostatic relationship between host, microbiome, and virome could be a factor in determining the efficacy of subsequent disease susceptibility, immunological responses, and long-term psychopathological effects of diseases that affect the CNS, including COVID-19 [170]. Probiotics can stimulate and modulate the immune system as well as reduce inflammation [171]. They act on the innate and adaptive immune system to decrease the severity of infections in the upper respiratory tract and the gastrointestinal tract [172].

**Figure 2:**
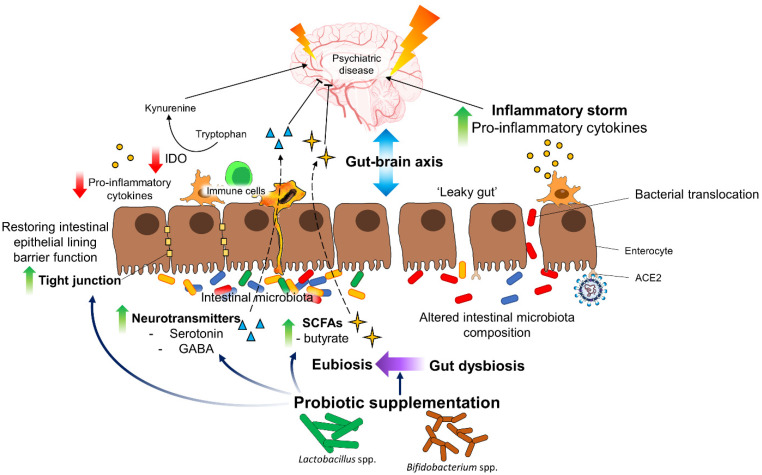
Proposed mechanisms of probiotics in the management of psychiatric symptoms in COVID-19 patients.

As mentioned earlier, cytokine dysregulation is associated with a psychiatric disorder and is elevated in COVID-19 patients. Furthermore, a cytokine storm occurs as proinflammatory cytokines increase, probably due to the T-helper cell (Th1) response in the lung tissue [172,173]. Thus, probiotics come into place in this context, as they have anti-inflammatory, antipathogenic, and antimicrobial properties, which aids the restoration and maintenance of intestinal homeostasis and microbial balance. They mediate its anti-inflammatory effects by modulating pro-inflammatory cytokines, regulating IDO activity, and restoring intestinal barrier function [165] (Figure 2). A recent meta-analysis revealed that probiotic intervention can significantly reduce the expression of IDO, an important enzyme which metabolizes tryptophan to kynurenine, in the immune cells and plasma of patients across several clinical trials [174]. Kynurenine and its metabolites play important roles in mediating inflammatory effects relevant to mood, anxiety, and psychotic disorders [175]. With that being said, probiotics can modulate the kynurenine pathway where the activation of IDO in response to inflammatory stimuli can be inhibited, hence, reducing inflammation-induced CNS pathology.

Probiotics and their metabolites, such as short-chain fatty acids (SCFAs), possess excellent potential in maintaining the integrity of the gut barrier by regulating the tight junctions between cells, limiting the translocation of gut bacteria across the intestinal barrier and reducing the activation of gut-associated immune cells. Thus, probiotics and SCFAs supplementation could potentially confer protection toward SARS-CoV-2 entry and trigger severe immunological changes in the gut. In the brain, SCFAs are also shown to reduce neuroinflammation by downregulating microglial activation and, thus, the pro-inflammatory cytokines secretion [176]. Furthermore, SCFAs also maintain the blood–brain barrier’s integrity by enhancing the expression of tight-junction proteins [177]. In addition to SCFAs, neurotransmitters such as serotonin and gamma-aminobutyric acid (GABA) are also metabolites produced by intestinal microbiota and probiotics. These neurotransmitters play major roles in orchestrating the normal functioning of the brain; imbalances in these neurotransmitters trigger stress, anxiety, depression, and impaired cognition. In fact, clinical studies have shown probiotic interventions improve and relieve anxiety and stress, and improve the mental status of depressed patients [168,178,179].

That being said, with regards to depression, recent research has found that certain classes of antidepressants potentially cause adverse effects due to their antibacterial properties and gut microbiota dysbiosis [180]. This suggests that maintaining a healthy gut microbiome is beneficial for healthy individuals, COVID-19 patients, and COVID-19 survivors having psychiatric sequelae. Probiotics may be one of the safest adjunctive treatments for alleviating psychiatric sequelae in post-COVID-19 survivors, as probiotics belong to the microbial genera commonly found in the intestinal tract. Therefore, compared to psychotropic medications, probiotics may have a lower risk of dependence, allergies [167], and lack conventional psychotropic medications’ side effects. They also reduce stigmatization [165], allowing for better the management of psychiatric symptoms and better overall health; in other words, killing two birds with one stone.

## 7. Conclusions

**Figure 3:**
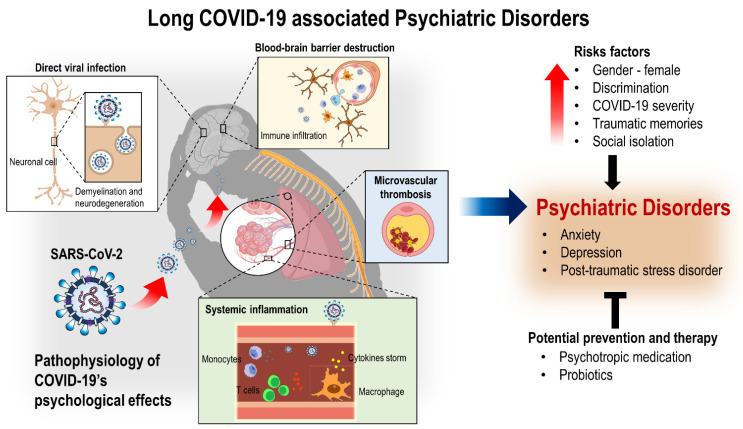
Illustration of long COVID-19-associated psychiatric disorders. It is known that COVID-19 psychologically affects patients, leading to psychiatric disorders such as anxiety, depression, and post-traumatic stress disorder. These symptoms can be potentially alleviated by probiotics, as they may be one of the safest adjunctive treatments for alleviating psychiatric sequelae in post COVID-19 survivors.

Although the long-term neuropsychiatric burden of COVID-19 is unknown, it is likely to be significant, as COVID-19’s mental health consequences have been shown to continue even after hospital discharge (Figure 3). SARS-CoV-2 causes physical illness and has a lasting psychological impact, especially for those severely affected and hospitalized. The exact cause of these psychological effects is yet to be determined, as it could be the direct action of the coronavirus on the brain and CNS, or indirect effects via systemic inflammatory responses to the virus, or a result of psychological stressors such as being infected, stigma, and the experience of being in the ICU. The consistent risk factors for psychiatric manifestations are female gender and history of psychiatric disorders.

In addition to concerns about the direct effects of psychiatric manifestations, it is important to note that delirium and stress-related symptoms (depression, anxiety, PTSD) were associated with approximately a four-times increase in the chance of developing neurocognitive impairments [78]. The persistent symptoms of long COVID-19 appear to affect cognitive and physical function, health-related quality of life, and participation in society [181]. For example, Bottemanne et al. found that depression after an acute COVID-19 episode might be linked to an increased risk of some persistent physical symptoms, including pain and dyspnea [182]. Hence, early interventions are crucial to combat the rising psychiatric manifestation of COVID-19 and to improve the functioning and quality of life of those affected, as well as to reduce the chances of developing neurocognitive impairments in addition to the already-debilitating psychiatric manifestations. Standard screening tools should be implemented to identify depression-, anxiety-, and PTSD-affected individuals. There should be neuropsychological evaluations for post-COVID-19 patients [10]. According to Bonizzato et al., there seems to be a need for an adequate assessment of cognitive, behavioral, and psychological variables in post-COVID-19 patients [183]. This is consistent with the findings by Gouraud et al., who found a robust link between cognitive complaints and psychological distress [184], and Poletti et al., who found that depression is the best predictor of cognitive performance and of its improvement [185]. Hence, further research is critical to further describe a more granular-level picture of the long-term psychiatric functioning post-COVID-19. Additionally, close interdisciplinary collaboration between health experts and specialized post-COVID-19 rehabilitation centers should be made available to better manage post-COVID-19 syndromes [186]. Interventions to decrease COVID-19-associated self-stigma and improving the mental health of patients could play a part to improve patients’ health-related quality of life [187]. Last but not least, rather than using conventional psychotropic medications alone, probiotics could be a safe adjunctive treatment for alleviating psychiatric sequelae in post COVID-19 survivors.

## Data Availability

Not applicable.
